# Pharmacological approaches of ADHD

**DOI:** 10.1192/j.eurpsy.2021.227

**Published:** 2021-08-13

**Authors:** R. Cooper, E. Williams, S. Seegobin, C. Tye, J. Kuntsi, P. Asherson

**Affiliations:** 1 Unit For Social And Community Psychiatry, East London NHS Foundation Trust/Queen Mary University of London, London, United Kingdom; 2 Social, Genetic And Developmental Psychiatry Centre, King’s College London, Institute of Psychiatry, Psychology and Neuroscience, London, United Kingdom; 3 Department Of Medical And Molecular Genetics, King’s College London, London, United Kingdom

## Abstract

**Abstract Body:**

Adults with ADHD describe self-medicating with cannabis. A small number of psychiatrists in the US prescribe cannabis medication for ADHD, despite there being no evidence from trials. The EMA-C trial (Experimental Medicine in ADHD-Cannabinoids) was a pilot randomised placebo-controlled experimental study of a cannabinoid medication, Sativex Oromucosal Spray, in 30 adults with ADHD. The primary outcome was cognitive performance and activity level using the QbTest. Secondary outcomes included ADHD and emotional lability (EL) symptoms. From 17.07.14-18.06.15, 30 participants were randomly assigned to the active (n=15) or placebo (n=15) group. For the primary outcome, no significant difference was found in the intent-to-treat analysis although the overall pattern of scores was such that the active group usually had scores that were better than the placebo group (Est=-0.17,95%CI-0.40-0.07, p=0.16, n=15/11 active/placebo). For secondary outcomes Sativex was associated with a nominally significant improvement in hyperactivity/impulsivity (p=0.03) and a cognitive measure of inhibition (p=0.05), and a trend towards improvement for inattention (p=0.10) and EL (p=0.11). Per-protocol effects were higher. Results did not meet significance following adjustment for multiple testing. One serious (muscular seizures/spasms) and three mild adverse events occurred in the active group and one serious (cardiovascular problems) adverse event in the placebo group. Adults with ADHD may represent a subgroup of individuals who experience a reduction of symptoms and no cognitive impairments following cannabinoid use. While not definitive, this study provides preliminary evidence supporting the self-medication theory of cannabis use in ADHD and the need for further studies of the endocannabinoid system in ADHD.

**Disclosure:**

During this work-RC was a Ph.D. student funded by a grant to PA from Vifor Pharma. PA received funds (consultancy/sponsored talks/research/education) from Shire, Lilly, Novartis, Janssen, PCMScientific, Vifor Pharma, QBTech. Sativex was free from GW Pharm

